# Quantitative analysis of optical coherence tomographic angiography (OCT-A) in patients with non-arteritic anterior ischemic optic neuropathy (NAION) corresponds to visual function

**DOI:** 10.1371/journal.pone.0199793

**Published:** 2018-06-28

**Authors:** Eric D. Gaier, Mengyu Wang, Aubrey L. Gilbert, Joseph F. Rizzo, Dean M. Cestari, John B. Miller

**Affiliations:** 1 Department of Ophthalmology, Massachusetts Eye and Ear Infirmary, Harvard Medical School, Boston, MA, United States of America; 2 Schepens Eye Research Institute, Harvard Medical School, Boston, MA, United States of America; Bascom Palmer Eye Institute, UNITED STATES

## Abstract

**Purpose:**

Non-arteritic anterior ischemic optic neuropathy (NAION) is the most common cause of non-glaucomatous optic neuropathy in older adults. Optical coherence tomographic angiography (OCT-A) is an emerging, non-invasive method to study the microvasculature of the posterior pole, including the optic nerve head. The goal of this study was to assess the vascular changes in the optic nerve head and peripapillary area associated with NAION using OCT-A.

**Design:**

Retrospective comparative case series.

**Methods:**

We performed OCT-A in 25 eyes (7 acute and 18 non-acute) in 19 patients with NAION. Fellow, unaffected eyes were analyzed for comparison. Patent macro- and microvascular densities were quantified in the papillary and peripapillary regions of unaffected, acutely affected, and non-acutely affected eyes and compared across these groups according to laminar segment and capillary sampling region, and with respect to performance on automated visual field testing.

**Results:**

In acutely affected eyes, OCT-A revealed a reduction in the signal from the major retinal vessels and dilation of patent superficial capillaries in the peripapillary area. By contrast, non-acutely affected eyes showed attenuation of patent capillaries. The peripapillary choriocapillaris was obscured by edema in acute cases, but was similar between non-acute and unaffected eyes. The degree of dilation of the superficial microvasculature in the acute phase and attenuation in the non-acute phase each correlated inversely with visual field performance. The region of reduced patent capillary density correlated with the location of visual field defects in 80% of acute cases and 80% of non-acute cases.

**Conclusions:**

OCT-A reveals a dynamic shift in the superficial capillary network of the optic nerve head with strong functional correlates in both the acute and non-acute phases of NAION. Further study may validate OCT-A as a useful adjunctive diagnostic tool in the evaluation of ischemic optic neuropathy.

## Introduction

Non-arteritic anterior ischemic optic neuropathy (NAION) is the most common cause of non-glaucomatous optic neuropathy among middle-aged and older adults and leads to irreversible visual loss [1,2]. Despite being relatively common and extensively studied, the pathogenesis of NAION remains elusive [3,4,5].

Systemic diseases that increase the risk for NAION include vascular diseases such as systemic hypertension and diabetes mellitus with end-organ damage [6,7,8]. Hypoperfusion of the optic nerve head, caused by nocturnal hypotension [9], obstructive sleep apnea [10], and erectile dysfunction medications [11], has been implicated in NAION. Vitreo-papillary traction has also been suggested as causative factor [12], but may instead cause a different type of optic neuropathy. The only clear anatomic risk factor for NAION is a small cup-to-disc ratio [13]. Moreover, the pathogenesis and pathophysiology of NAION continues to be debated, but it is generally understood to include ischemia.

The use of optical coherence tomography (OCT) is becoming more common in research and the clinical management of patients with optic neuropathies, including NAION [14]. For example, spectral-domain OCT has helped elucidate the relative degree of involvement for each optic disc sector with respect to retinal nerve fiber layer swelling and functional loss in acute and non-acute phases of NAION, respectively [15,16]. OCT-Angiography (OCT-A) is a non-invasive modality that provides a laminar analysis of the microvasculature of the posterior pole within the retina and choroid, including at least the pre-laminar region of the optic disc [17]. This technique would thus seem to be ideal to study vascular pathology in a disorder like NAION, which has been thought to be ischemic in nature. Only a few studies, with small numbers of patients in isolated phases of the disease, have employed OCT-A to evaluate microvascular changes in NAION [18,19,20,21,22,23].

In this comparative case series, we employed this recently developed imaging modality to conduct the first objectively quantitative study distinguishing the macro- (major retinal vessels) and micro-vascular (capillary) changes associated with both the acute and non-acute phases of NAION.

## Methods

### Patients

**Fig 1:**
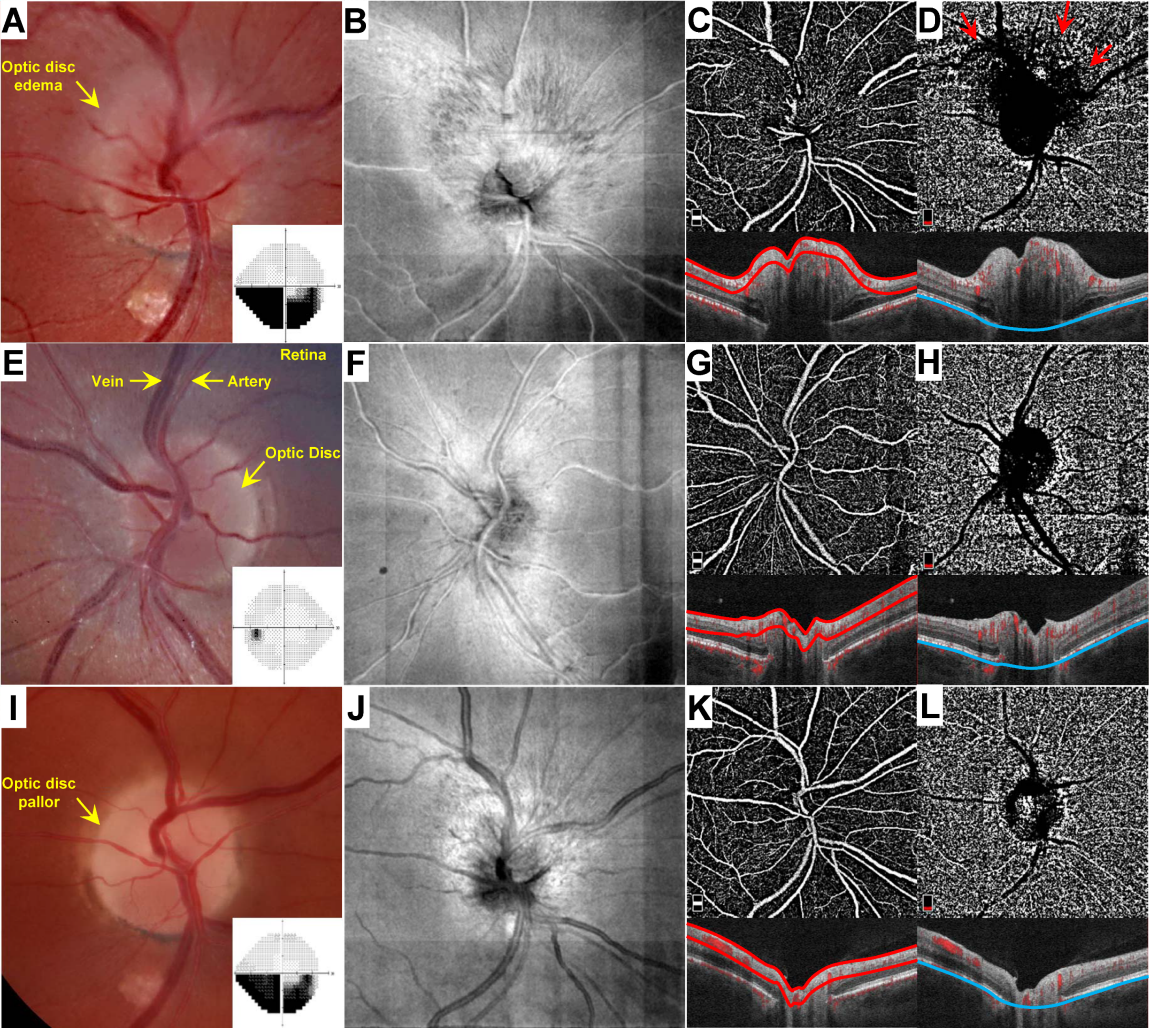
Representative OCT-A analysis in a patient with unilateral NAION. The patient is a 42 year-old man who presented 4 days after experiencing painless visual loss in the right eye. Fundus photographs of the affected right (A) and unaffected left (E) eyes. Automated (Humphrey 24–2) perimetry grey scale results for the right (A) and left (E) eyes are provided as insets. En face images of the right (B) and left (F) eyes. OCT-A images with segmentation of the superficial papillary capillary network (C, G) and choriocapillaris (D,H). Corresponding B-scan images denote segmentation boundaries (red, superficial; blue, choriocapillaris). Red arrows in D denote blockage artifact imparted by overlying optic disc edema. (I-L) Evaluation of the same patient 7 weeks after the initial presentation. (I) Fundus photographs of the affected right eye. Inset depicts Automated (Humphrey 24–2) perimetry result for the right eye at that time. (J) En face image of the affected optic disc at that time. OCT-A image with segmentation of the superficial papillary capillary network (K) and choriocapillaris (L).

The Massachusetts Eye and Ear Institutional Review Board approved this study, which adhered to the declaration of Helsinki, and waved the need for informed consent. Patients who presented to the Massachusetts Eye and Ear Infirmary Neuro-Ophthalmology service with NAION who had OCT-A imaging between February 1, 2016 and May 31, 2017 were included. Patients were retrospectively identified from an internally maintained OCT-A imaging database, and were categorized as either acute or non-acute based on the presence or absence of optic disc edema, respectively, at the clinical examination. Optic disc edema with or without hemorrhage were readily apparent in all acute cases and confirmed by spectral-domain OCT (**Fig 1A–1D**). Fellow, unaffected eyes were also evaluated and imaged by OCT-A at the initial presentation (**Fig 1E–1H**). Patients typically had a history of sudden, painless, monocular vision loss. Neuro-imaging was not obtained for typical acute presentations; for non-acute patients with a history suggestive of NAION, patients were not included unless an MRI had confirmed the absence of compressive lesion. All acute cases subsequently progressed to optic disc pallor over weeks (**Fig 1I–1L**). Three patients who underwent OCT-A in the acute phase were also re-imaged at their follow-up examination, and those eyes affected eyes were included in the non-acute phase group in addition to the acute phase group (**Fig 1A–1D and 1I–1L**). Patients with superimposed vascular pathology (e.g. diabetic retinopathy, venous occlusion) were excluded from the study. Poor OCT-A image quality precluded inclusion of the affected eye in three patients, nonetheless the fellow eyes of these patients were included as part of the unaffected (control) group.

### Clinical features

Mean visual acuities were 0.2 ± 0.3 LogMAR (Snellen equivalent: 20/32 ± 3 lines, range: 20/15-20/125) for acutely affected eyes, and 0.4 ± 0.6 LogMAR (Snellen equivalent: 20/40 ± 6 lines, range: 20/15-counting fingers) for non-acutely affected eyes. In cases except for sequential presentations, a relative afferent pupillary defect was noted in 5/6 acute cases and 10/13 non-acute cases. Dyschromatopsia was evident on Ishihara color plate testing in 3/7 acute cases and 4/15 non-acute cases of patients who were able to identify the control plate. The mean visual acuity across all unaffected, fellow eyes was 0.0 ± 0.1 LogMAR (Snellen equivalent: 20/20 ± 1 line). No unaffected fellow eyes exhibited a relative afferent pupillary defect or dyschromatopsia.

### Image acquisition

In all cases, OCT-A images were obtained using the Optovue Avanti (Fremont, CA) and analyzed using ReVue software. Two imaging windows (4.5x4.5 mm and 6x6 mm) were obtained in most cases, and the lowest power high-quality image was used for quantitative analysis by default to optimize complete sampling of the peripapillary region. Automated laminar segmentation provided by the ReVue software program was manually adjusted as necessary. All choriocapillaris segmentation images required manual adjustment to move the sampling region below the retinal pigment epithelium as described by Wright Mayes, et al. (2017).[23]

### Quantification of patent macro- and micro-vascular densities

**Fig 2:**
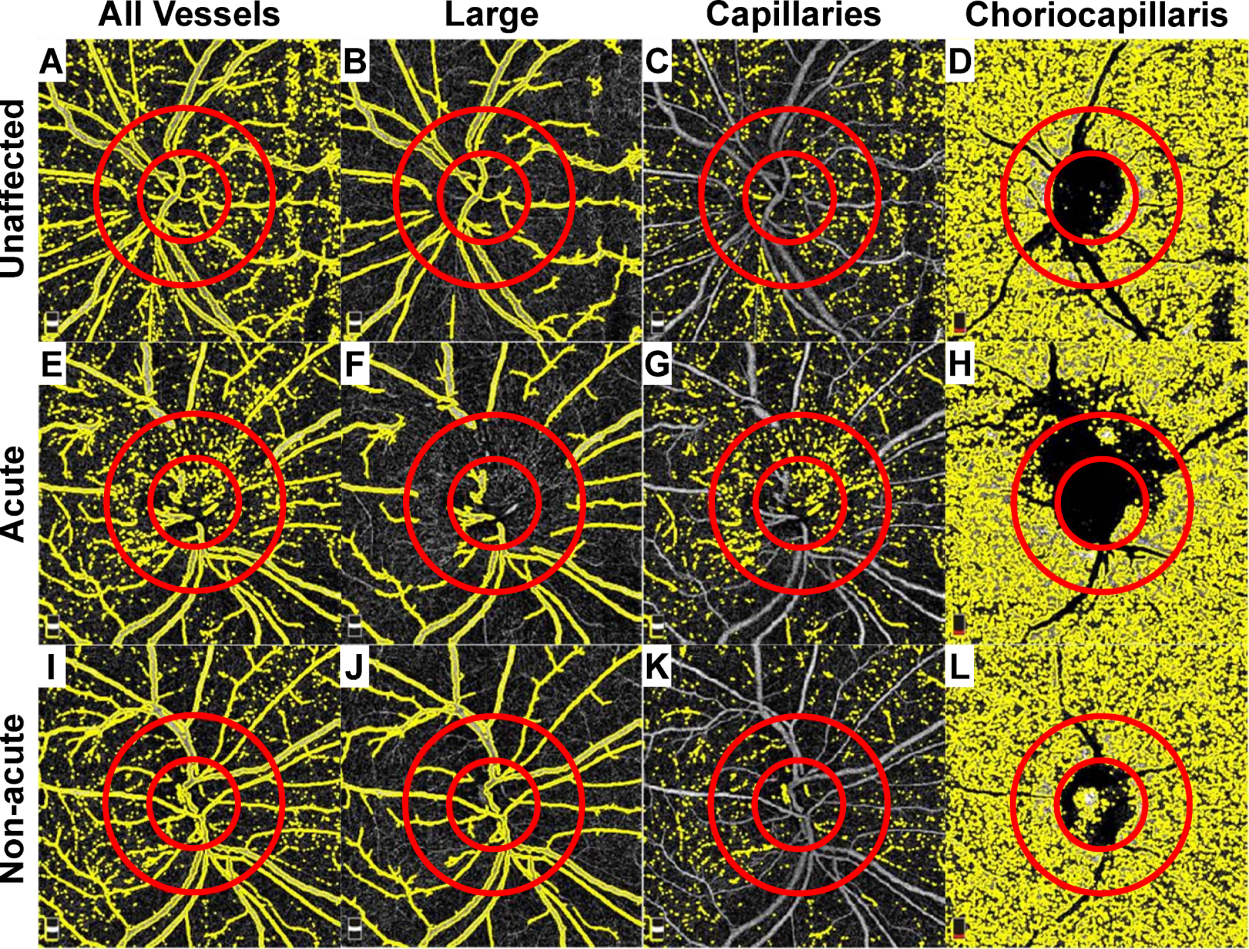
Quantitative analysis of OCT-A images. The superficial and choriocapillaris segmentation images from **Fig 1** are included. In all cases, a 1.73 mm diameter region of interest (ROI) was centered on the optic disc using the deep choroidal images as a reference for the disc margins (smaller red circle). Another 3.46 mm diameter ROI was drawn to sample the peripapillary capillary network (larger red circle). The total angiographic signal (A, E, I), filtered large vessels (B, F, J), superficial capillaries (C, G, K) and choriocapillaris segmentation are included for the unaffected (A-D), acutely affect (E-H), and non-acutely affected (I-L) eyes.

We imaged and separately quantified the superficial macrovasculature (major retinal vessels) and microvasculature (capillaries), and the choriocapillaris (**Fig 2**). An automated image processing algorithm was developed to quantify the superior and inferior patent capillary densities within the optic disc and within the peripapillary region. The optic disc center was marked by an experienced trained observer (MW) who was masked to clinical group with customized software developed by our group using MATLAB (MathWorks, Natick, MA). A circle with a radius of 0.85 mm was used to define the region of the optic disc. The annular region between this circle and a second circle with a radius of 1.73 mm was specified as the peripapillary region. A 2D median filter (1 pixel x 1 pixel) was applied to remove noise. Then, all vessels were segmented by thresholding the image with Otsu's method (**Fig 2A, 2E and 2I**) [24]. Large vessels (**Fig 2B, 2F and 2J**) and capillaries (**Fig 2C, 2G and 2K**) were defined as vessels with contiguous signal areas ≥50 μm^2^ and <50 μm^2^, respectively. Parameters for defining vessel caliber categories were empirically optimized to segregate based on visibility on standard fundus photography. The total area within the sampling region occupied by either large or small caliber angiographic signal was quantified as a fraction of the region of interest, and this metric was used for all comparative analyses.

The papillary angiographic signal generated by the choriocapillaris laminar segmentation was highly variable and could not be reliably extracted, and therefore it was not included in the analysis (**Fig 2D, 2H and 2L**). Among eyes that had presented in the acute phase of NAION, edema of the peripapillary region blocked signal acquisition from the choriocapillaris and precluded reliable measurements for comparisons (**Fig 2H**), so this parameter also was not analyzed. The total angiographic signal in the peripapillary region of the choriocapillaris was used for all comparative analyses in the non-acute phase only (**Fig 2L**). Raw data can be found in **S1 Data**.

### Visual field testing

Automated (Humphrey, 24–2 SITA standard) perimetry was performed on the day of OCT-A analysis in all cases. Data was not included if the reliability parameters provided by the test paradigm exceeded 30% fixation losses, 20% false positives, or 20% false negatives. Mean and pattern deviation results provided on the Humphrey perimeter report were compared with angiographic vessel densities. Hemi-field values were calculated by averaging the deviations from normal for all tests points in the superior and inferior hemi-fields of the 24–2 plot for each eye. Raw data can be found in **S1 Data**.

### Statistical analysis

Comparisons among unaffected eyes, acutely affected and non-acutely affected eye with NAION were conducted by one-way ANOVA within each OCT-A sampling area (i.e. disc, peripapillary, and total) using a Bonferroni correction for multiple comparisons. Pearson correlation (one-tailed) was used to test hypothesized (*a priori*) relationships as specified between OCT-A signal densities and visual field parameters. The relationship between patent peripapillary choriocapillary signal and visual field performance was tested using a two-tailed analysis. Spearman rank correlation (two-tailed) was used to test relationships between OCT-A signal densities and LogMAR visual acuities. In all cases *p*<0.05 was considered statistically significant.

## Results

Twenty four patients (19 men and 5 women) were included in this study. Of the 25 eyes with NAION, 7 eyes from 7 patients were imaged in the acute phase (one patient had experienced sequential NAION with acute involvement of the fellow eye) (**S1 Table**), and 18 eyes from 16 patients in the non-acute phase (3 eyes from 3 patients in this group represented follow-up testing from the acute group). Patients were aged 59.5 ± 9.2 years (acute: 58.7 ± 13.2 years, non-acute: 59.2 ± 7.8 years; *p* = 0.905). The time between onset of visual symptoms and presentation was 12.5 ± 17.6 days (range: 2–48 days) for acute eyes and 1.8 ± 3.4 years (range: 28 days -10.0 years) for non-acute eyes. Sixteen unaffected (fellow) eyes were also analyzed by OCT-A using the same quantitative method for comparison with acute and non-acute NAION conditions.

### Quantitative OCT-A analysis in acute and non-acute NAION

**Fig 3:**
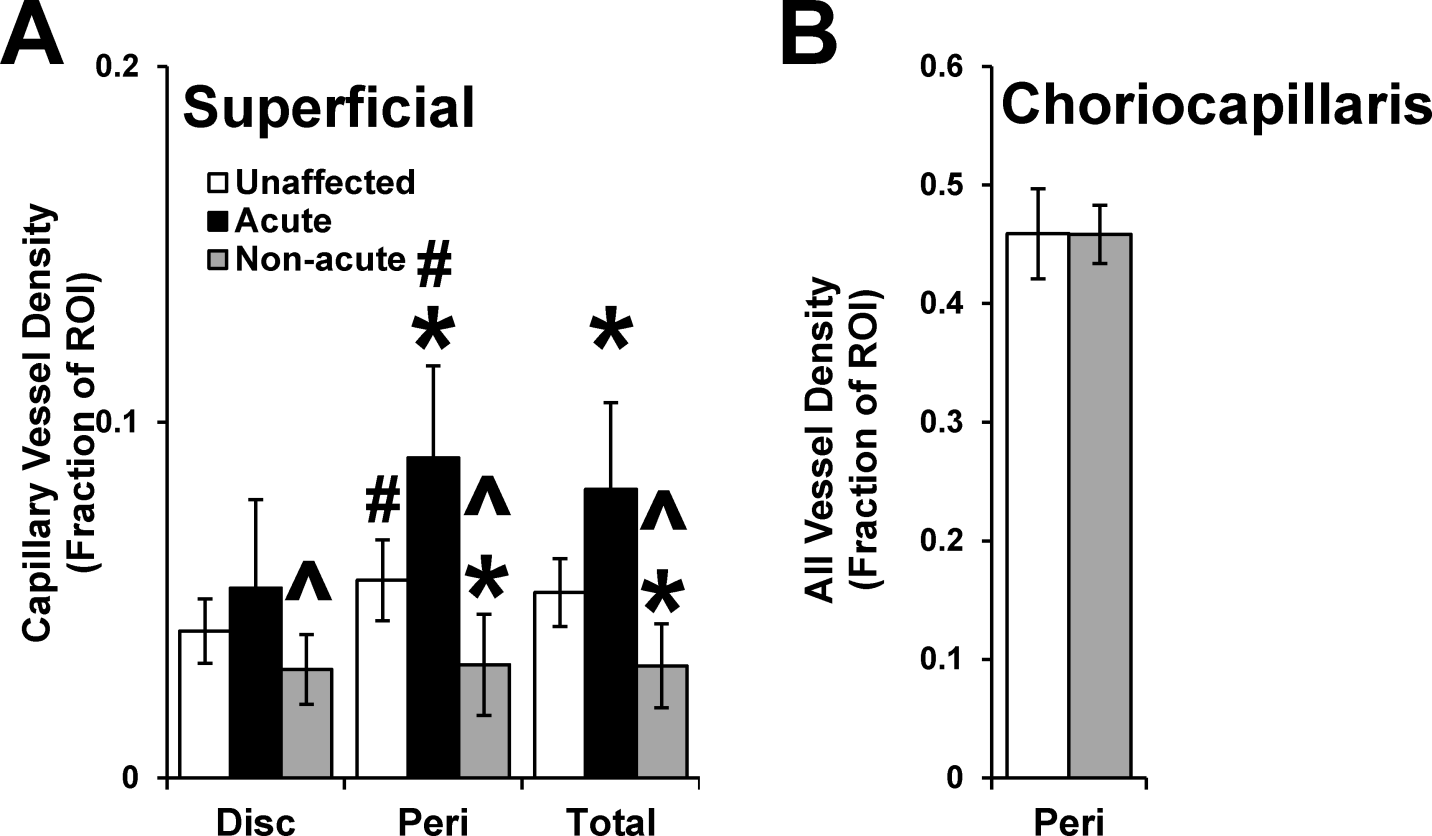
Quantitative analysis of OCT-A data and comparisons across the NAION disease course. (A) The superficial patent capillary density was quantified and compared between unaffected, acutely affected and non-acutely affected eyes. (B) The patent choriocapillary density was quantified and compared between unaffected and non-acutely affected eyes. Error bars represent standard deviation. * denotes *p*<0.05 versus unaffected; ^ denotes *p*<0.05 versus the acute group; # denotes *p*<0.05 versus disc.

OCT-A images were quantitatively analyzed by separately sampling the angiographic signal corresponding to the major retinal vessels, the superficial capillary network overlying the optic disc and in the surrounding peripapillary region, and the peripapillary choriocapillaris in a systematic fashion for all affected and unaffected eyes (**Fig 2A–2L**). In unaffected eyes, the OCT-A signal in the superficial lamina comprised by the major retinal vessels was approximately 4-5-fold greater than that contributed by the superficial capillaries in both the disc and peripapillary sampling regions (**Fig 3A** versus **S1 Fig**). There was a greater patent capillary vessel density in the peripapillary region compared to the papillary region in unaffected and acute eyes (*p* values<0.005), but not for non-acutely affected eyes (*p* = 0.745) (**Fig 3A**).

In eyes acutely affected by NAION, superficial segmentation showed partial obscuration of the OCT-A signal from the large retinal vessels at the disc margin (**Fig 1C**). By quantitative analysis, there was a significant reduction in the angiographic signal in acutely affected eyes compared to unaffected eyes in the papillary and peripapillary regions (Bonferroni-corrected individual comparisons: *p* values<0.022; **S1 Fig**). There was no difference in the major vessel density between non-acutely affected and unaffected eyes in both sampling regions (Bonferroni-corrected individual comparisons: *p* values>0.209).

OCT-A revealed that the superficial patent capillary network was dilated in all acutely affected eyes. Quantitative analysis showed a significant increase in the superficial patent capillary density among acute compared to unaffected eyes in the peripapillary region (*p*<0.001), but did not reach statistical significance in the papillary sampling region (*p* = 0.150) (**Fig 3A**). This increase remained statistically significant when the papillary and peripapillary sampling regions were combined (*p*<0.001) (**Fig 3A**; total). By contrast, OCT-A revealed the superficial patent capillary network to be attenuated on average in non-acutely affected eyes. Quantitative analysis showed a significant reduction in the superficial patent capillary density among non-acute compared to unaffected eyes with NAION in the peripapillary region (*p*<0.001), but did not reach statistical significance in the papillary sampling region (*p* = 0.066; **Fig 3A**). There was a significant difference between the patent capillary density overlying the disc in acutely and non-acutely affected eyes (*p* = 0.001). Non-acutely affected eyes also exhibited a significant reduction in the peripapillary patent capillary density compared to unaffected and acutely affected eyes (*p* values<0.001). This reduction remained statistically significant when the disc and peripapillary sampling regions were combined (*p* values<0.001; **Fig 3A**; total). Together, these data demonstrate reciprocal changes to the superficial patent capillary density overlying and around the optic nerve in patients acute and non-acute NAION.

The OCT-A signal of the choriocapillaris was approximately 10-fold denser compared to that of the superficial capillaries within both affected and unaffected groups, representing the established anatomic capillary density and flow in this structure. OCT-A analysis of the choriocapillaris showed no difference in the angiographic signal within the peripapillary region between non-acutely affected and unaffected eyes (*p* = 1.000) (**Fig 3B**).

### Comparative analysis of OCT-A results and visual field testing performance

**Fig 4:**
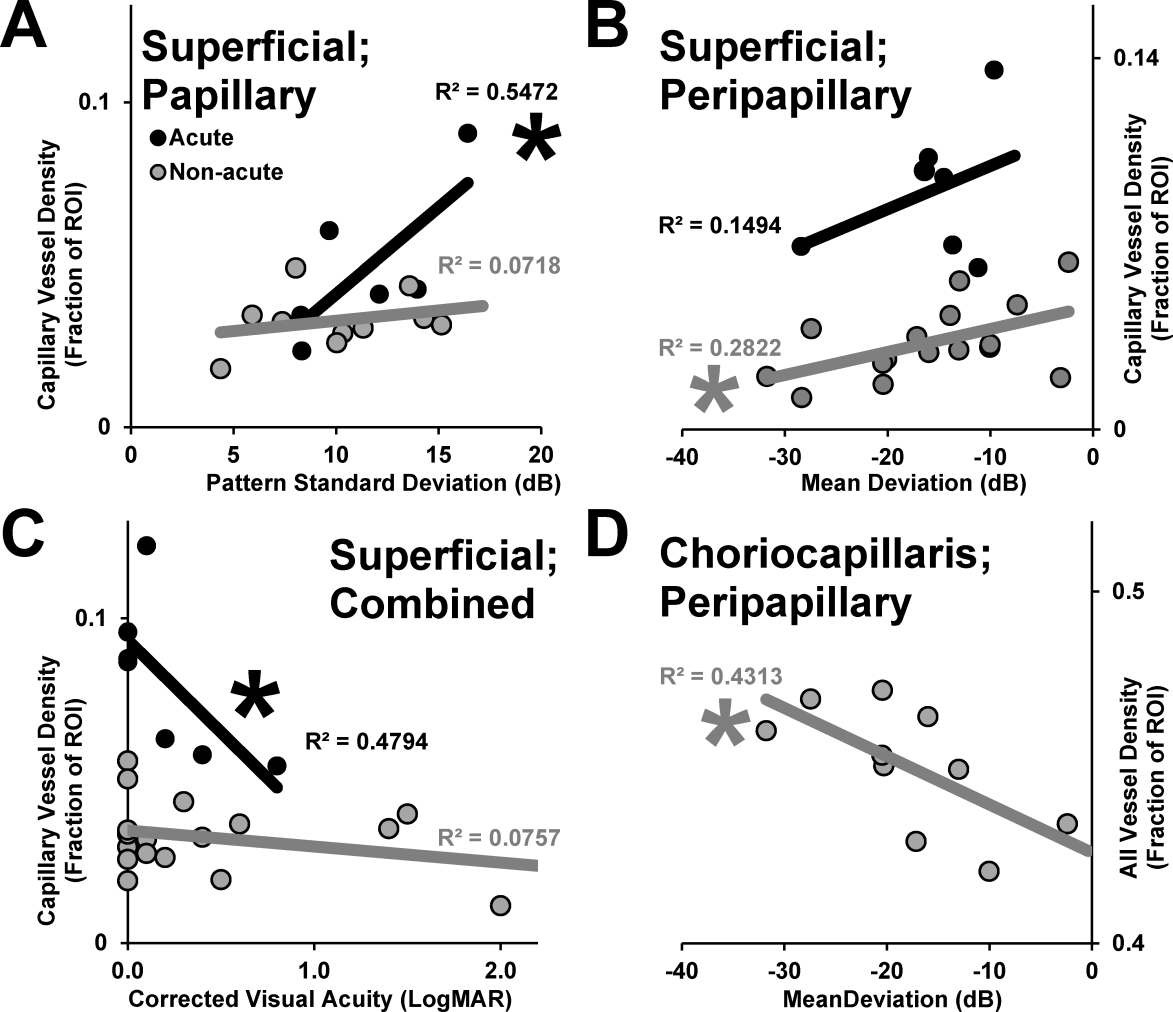
Relationships between patent capillary density assessed by OCT-A and visual function. (A) Superficial patent capillary density within the optic disc is plotted according to pattern standard deviation among eyes with acute and non-acute NAION. (B) Superficial patent capillary density in the peripapillary area is plotted according to mean deviation among eyes with acute and non-acute NAION. (C) The superficial patent capillary density in the total sampling area is plotted according to best corrected visual acuity (LogMAR). (D) The total patent choriocapillary angiographic signal measured in the peripapillary region for non-acutely affected eyes with NAION. Trend lines represent linear best fit, and R^2^ values are provided for acutely and non-acutely affected eyes according to shade. * denotes significant correlation (*p*<0.05).

The regional patent capillary densities from OCT-A imaging were compared according to visual field testing performance. We hypothesized that deficits in visual field testing performance would mirror the reciprocal changes we observed in superficial patent capillary densities on OCT-A. Among acutely affected eyes, greater superficial patent capillary density within the optic disc, but not peripapillary region (*p* = 0.164) (not shown), correlated significantly with worse visual field performance as assessed by pattern deviation (*p* = 0.046) (**Fig 4A**). By contrast, there was no such statistically significant relationship among non-acutely affected eyes between the patent capillary density in the papillary or peripapillary regions and pattern deviation (*p* values>0.227). Among non-acutely affected eyes, lower peripapillary, but not papillary (*p* = 0.089), superficial patent capillary density correlated significantly with worse visual field performance as assessed by mean deviation (*p* = 0.014) (**Fig 4B**). There was no corresponding relationship among acutely affected eyes between the combined patent capillary density and mean deviation (*p* = 0.437).

Among acutely affected eyes, lower total superficial patent capillary density correlated significantly with worse visual acuity (*p* = 0.041) (**Fig 4C**). There was no corresponding relationship among non-acutely affected eyes between total superficial patent capillary density and visual acuity (*p* = 0.913). There was no statistically significant relationship in either direction between visual field performance parameters and visual acuity in acutely affected eyes (*p* values>0.425) (not shown). Among non-acutely affected eyes, greater patent choriocapillary vessel density correlated significantly with worse visual field performance as assessed by mean deviation (*p* = 0.033) (**Fig 4D**). Projection artifact imparted by the major retinal vessels did not account for this relationship because there was no relationship between major vessel density and mean deviation in the peripapillary region (**S2 Fig**).

**Fig 5:**
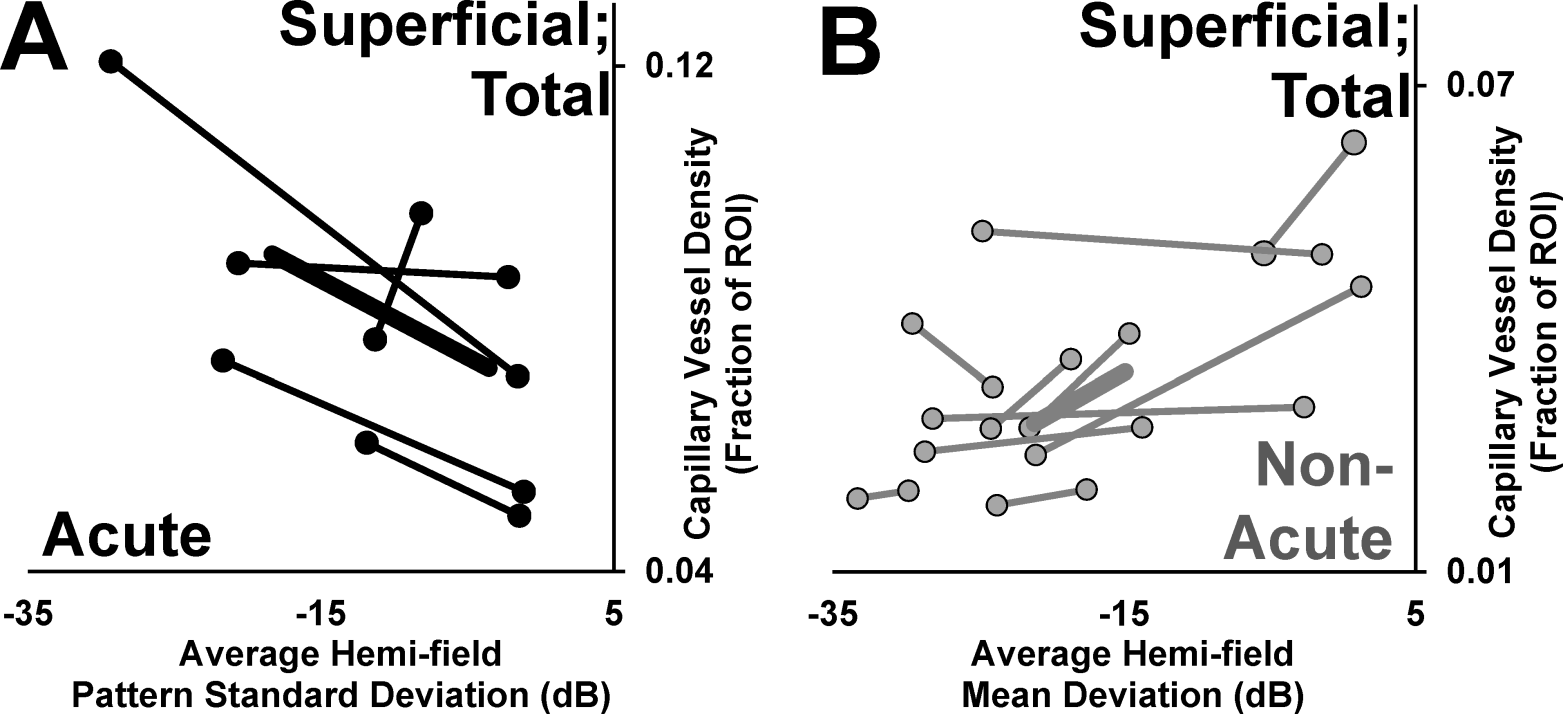
Analysis of sectoral vessel density and hemifield correspondence. The superior and inferior sectors of the combined optic disc and peripapillary regions were paired the corresponding inferior and superior mean visual field deviations. (A) The superior and inferior combined sampling areas are plotted according to hemifield pattern standard deviation for acutely affected eyes. (B) The superior and inferior combined sampling areas are plotted according to hemifield mean deviation for non-acutely affected eyes. Points represent each sector-hemifield pairing and lines connect each half of the affected eyes. Thick trend lines represent the mean relationship between the more severely affected hemidisc sector and the less severely affected hemidisc sector within eyes.

To test for correspondence between these microvascular changes and visual function, we divided the papillary and peripapillary regions sampled into superior and inferior sectors for comparison with average mean and pattern standard deviations in each associated hemi-field. Among acutely affected eyes with reliable visual field testing, higher superficial patent capillary density was associated with worse hemi-field pattern standard deviation performance in 4/5 (80%) eyes (**Fig 5A**). Conversely, among non-acutely affected eyes with reliable visual field testing, lower superficial patent capillary density was associated with worse hemi-field mean deviation performance in 8/10 (80%) eyes (**Fig 5B**).

## Discussion

The etiology of NAION is not known, but the condition is believed to be caused by ischemia of retinal ganglion cell axons in the region of the laminar cribrosa [5]. Toward the goal of developing a better understanding of the vascular pathophysiology, we applied a recently developed, non-invasive modality for studying the superficial microvasculature of the optic nerve head in eyes affected by NAION. The most significant finding of this work was the demonstration of bidirectional changes in superficial patent capillary density associated with the optic nerve head in the natural course of NAION (**Table 1**). The significant correlation between these microvascular changes and regional visual deficits represents an important structure-function relationship and underscores the clinical importance of microvascular dynamics in NAION.

**Table 1:** Summary of OCT-A results for NAION.

	Major Retinal Vessels	Superficial Capillaries	Choriocapillaris
**Acute**	↓	↑^a^	NA
**Non-Acute**	↔	↓^b^	↔^c^

a. Associated with worse pattern deviation and better visual acuity. Affected hemi-field correspondence.

b. Associated with worse mean deviation. Affected hemi-field correspondence.

c. Associated with worse mean deviation.

The vaso-occlusive hypothesis for the pathogenesis of NAION is supported by strongly associated systemic vascular risk factors [6,7,8], the acute nature of onset, prominent vascular findings on presentation, and configuration of vascular anatomy at the optic nerve head [5]. The superficial microvascular dilation we observed in the current study is most likely an epiphenomenon, possibly representing autoregulatory vasodilation to compensate for infarcted axons deeper within the optic nerve, posterior to where we are able to image with this method. Alternatively, congestion and crowding imparted by axonal swelling could lead to local venous outflow obstruction and/or possible shunting. Our data demonstrate a direct relationship between the angiographic capillary signal and visual field loss, but do not distinguish between these hypothetical mechanisms. A higher degree of optic nerve ischemia that in turn leads to greater visual loss is known to produce greater optic nerve head edema in the acute phase of an animal model of ischemic optic neuropathy [25], and it seems intuitive that associated vascular changes would follow the same pattern. Vascular attenuation in the non-acute phase likely represents pruning of capillaries in the setting of nerve fiber layer atrophy. The degree to which the microvasculature both expanded in the acute phase and attenuated in the non-acute phase corresponded to different visual field testing parameters (namely pattern deviation and mean deviation, respectively) (**Table 1**). In the acute phase, it may be that microvascular expansion is linked to the severity of axonal ischemia, whereas in the non-acute phase, attenuation is linked to the breadth of axons affected.

We found a dichotomy between the pattern deviation on visual field testing and best corrected visual acuity as functions of superficial microvascular dilation in the acute phase; higher density was associated with poorer visual field performance, but better visual acuity (**Table 1**). One could interpret superficial patent capillary dilation as protective for the papillomacular bundle fibers that originate in the fovea and perifovea. One might have expected to find an inverse relationship between pattern deviation and visual acuity; that no such effect was seen supports a central role for vascular dynamics that separately relates to these distinct visual areas in NAION.

Few prior studies have examined eyes with acute NAION using OCT-A, and all reported peripapillary vascular attenuation associated with the acute phase of NAION (**Table 2**) [21,22,23]. Two studies were qualitative; one with only a single patient with mild optic disc edema (compared to the acutely affected patients in our study) [23], and the other was performed with a different device (Cirrus HD-OCT with AngioPlex, Carl Zeiss Meditec, Dublin, CA) and showed only select examples of the 10 patients in their cohort and did not include fundus photography [21]. Sharma et al (2017) reported on 5 Chinese patients with NAION presenting within 7 days of symptom onset and quantitatively showed reductions in microvascular flow in the superficial and choroidal peripapillary regions [22]. Distinguishing the microvasculature from the major retinal vessels is likely the main contributor to disagreement with our results. In our study, we found that ~80% of the vascular density in the peripapillary region of normal eyes is attributable to the major retinal vessels (**Fig 3A** versus **S1 Fig**), and that signal drops by ~50% in the acute phase of NAION (**Fig 3A**) due to obscuration by edema (**Fig 1A–1C**). In addition, the high degree of hemorrhage in the examples provided in Sharma et al (2017) could produce considerable focal blockage that would reduce the quantified vessel density in a given sampling area. The example OCT-A images provided demonstrate expansion of the small caliber vessels overlying the optic disc and in the peripapillary patent capillary network, similar to what we observed and selectively quantified [22]. This comparison demonstrates the potential hazards of solely relying on automated software for quantification and how subtleties in methods can have significant impacts on results. This observation also demonstrates not only how a new technology provides new insights into an enigmatic disease, but how the disease provides insights into the limitations as well as the strengths of a technology.

**Table 2:** Summary of NAION OCT-A studies for NAION to date.

Study	Patients (N; not including control)	Acute (N; eyes)	Non-acute (N; eyes)	OCT-A device	Qualitative or Quantitative	Major Findings
Gaier et al 2017	19	7	18 (3 follow up)	Optovue RTVue-XR Avanti (Fremont, CA)	Quantitative	Acute: superficial microvascular dilation Non-acute: superficial microvascular attenuation. Correlation and correspondance with visual field.
Liu et al 2017	13	0	13	Optovue RTVue-XR Avanti (Fremont, CA)	Quantitative	Reduced peripapillary vessel density, but similar parafoveal vessel density compared with age-matched controls. Correlation with RNFL measurements.
Wright Mayes et al 2017	9	1	8	Optovue RTVue-XR Avanti (Fremont, CA)	Qualitative	Superficial microvascular and choriocapillaris attenuation with correspondence to visual field and RNFL/GCC.
Rougier et al 2017	10	10	0	Cirrus HD-OCT with Angioplex, Carl Zeiss Meditec, Dublin, CA	Qualitative	Focal disappearance of the superficial capillary radial pattern. No correlation with visual field.
Sharma et al 2017	5	6	3 (3 follow up)	Optovue RTVue XR 100 (Fremont, CA)	Quantitative	Global reduction of mean peripapillary flow density compared to age-matched controls; and mild relative improvement on follow up.
Hata et al 2017	15	0	15	Optovue (unspecified)	Quantitative	Reduction in vessel density in the peripapillary retina and within the optic disc. Correlation between peripapillary vessel density and visual field/RNFL, but not correspondence.

**Abbreviations:** RNFL, retinal nerve fiber layer; GCC, ganglion cell complex

We separated the superior and inferior hemi-disc to analyze sectoral associations within the same eye between OCT-A signal and visual dysfunction because the pattern of injury and visual dysfunction is often altitudinal in NAION. However, it is important to acknowledge that this pattern does not fit all cases. For example, Wright Mayes et al. (2017) showed extensive patterns of patent capillary attenuation extending to ¾ of the circumferential optic disc [23]. By contrast, the example we provide shows focal superficial patent capillary attenuation concentrated directly superior to the optic disc (**Figs 1 and 2**). Nevertheless, our analysis yielded a clear relationship between the relative involvement of each hemi-disc (superior versus inferior) and the corresponding hemi-field within acutely (**Fig 5A**) and non-acutely (**Fig 5B**) affected eyes independently in most cases (80%). This further supports the conclusion that sequential increase and decrease in OCT-A signal in the superficial peripapillary region corresponds to functional visual impairment in acute and non-acute phases of NAION, respectively.

Four studies have assessed microvascular changes in non-acute NAION using OCT-A (**Table 2**) [18,20,22,23]. Two quantitative studies, also using Optovue devices, reported microvascular attenuation in the non-acute phase of NAION with correlation and/or spatial correspondence between areas of vascular attenuation and RNFL thickness or visual field loss [18,20]. Similar findings have been described in glaucomatous optic neuropathy [26], and may reflect nerve fiber layer atrophy and secondary pruning of unnecessary capillaries in both cases. Our experience with the automated segmentation of the choriocapillaris using Optovue software supports the findings of Wright Mayes et al (2017) of a necessity of manual adjustment, which we applied identically in our study [23]. Two groups described flow impairments in the peripapillary choriocapillaris that we did not observe in the current study, even qualitatively. Despite the lack of difference in peripapillary patent choriocapillary vessel density, we demonstrated an association between higher patent choriocapillary vessel density and poor visual field performance (**Fig 4D**) that was not accounted for by blockage from the larger vessels (**S2 Fig**). This relationship may represent enhanced penetration and visualization of deep vascular structures when the overlying retina is thin and the microvasculature is attenuated. This could serve as another example of how a disease process can inform us about an applied technology.

There are some limitations of our study. First, we had longitudinal OCT-A imaging in both the acute and non-acute phases for 3 patients; ideally, one would make pair-wise comparisons to study disease course but this was an insufficient number to do so. Many acute NAION patients were not reimaged with OCT-A because they were enrolled into a clinical trial (NCT02341560) for an injectable medication, of which the effects on retinal and optic nerve head vasculature are unclear and could have potentially confounded our results. However, our relatively high numbers allowed us to uncover statistically significant relationships supported by the qualitative interpretations of those 3 patients, so it is unlikely that the unpaired analysis affected our findings or conclusions. Second, overlying edema and hemorrhage could produce a blockage artifact that could affect the quantification of angiographic signal in the sampling region. Furthermore, severe visual impairment would be expected to limit fixation and in turn degrade image quality, potentially introducing a bias toward exclusion of more severe cases. If either of these factors were playing a significant role, they would be in the direction of limiting the increase we observed in peripapillary microvasculature in the acute phase of NAION. That we nevertheless observed a statistically significant increase speaks to the magnitude of the effect of NAION on microvascular anatomy in the acute phase (**Fig 3**). Lastly, it is important to recognize that the “unaffected” eye group in our study should not necessarily be considered normal. Between 15% and 24% of the eyes in this group will go on to develop NAION in the 5 years following their initial presentation.[27] An important future direction will be to compare unaffected eyes in patients with NAION to eyes of patients without NAION looking for potential anatomic vascular markers in the optic nerve head that can help predict which eyes will go on to develop NAION.

OCT-A holds clear advantages over conventional OCT and fluorescein angiography. First, OCT-A is non-invasive and carries no risk of adverse reactions to intravenous dye. OCT-A has significant higher resolution with the capability of resolving the superficial papillary and retinal vasculature with micrometer precision [28]. This allows OCT-A imaging to be systematic, allowing for quantitative analysis and comparisons across study groups. OCT-A carries some limitations for analysis of NAION, especially in the acute phase. One disadvantage of OCT-A compared to FA is that OCT-A is not dynamic, so assessment of transit time in evaluation of venous occlusion, which shares considerable clinical overlap and is a risk factor for NAION,[6] is not possible. Second, all 7 cases of acute NAION in our study showed optic disc edema that imparted varying degrees of blockage artifact that was significant in all cases. Swept-source OCT-A, with enhanced depth of penetration and resolution through media opacities, potentially cotton wool spots and swelling of the nerve fiber layer, could help address some of the limitations observed in our study [29,30]. Aside from imparting a blockage effect, optic disc edema and distortion of the normal contour of the posterior pole challenges the fitting algorithm for laminar analysis. The Optovue software used in this study has a feature that allows for manual alterations for targeted laminar analysis, which was necessary to accurately study the choriocapillaris with this device [23].

Overall, the results presented in our study demonstrate the utility of OCT-A in advancing our understanding of the pathophysiology of NAION. Longitudinal analysis at serial time points in the disease course could provide major contributions to advance our understanding of this enigmatic disease. Furthermore, well characterized distinct features of NAION on OCT-A could facilitate prognostication and help guide enrollment in clinical trials or future therapeutics and improve our ability to counsel patients.

## Supporting information

S1 Table Acute NAION cases. Details regarding the age, gender, onset, visual acuity and automated perimetry performance indices are provided for the 7 patients presenting with acute NAION.
(DOCX)

S1 Fig Quantitative analysis of large vessel density measured by OCT-A and comparisons across the NAION disease course. Large, major retinal vessel density was quantified within superficial segmentation lamina and compared between unaffected, acutely affected and non-acutely affected eyes. Error bars represent standard deviation. * denotes *p*<0.05 versus unaffected; ^ denotes *p*<0.05 versus the acute group; # denotes *p*<0.05 versus disc.
(TIFF)

S2 Fig There is no relationship between patent choriocapillary density and visual field mean deviation. Large, major retinal vessel density in the peripapillary region is plotted according to mean deviation among eyes with non-acute NAION. The trend line represents the linear best fit, and the R^2^ value is provided.
(TIFF)

S1 Data Raw data for OCT-A quantitation and automated perimetry. (XLS)
