# Supplementary material for: Quantitative analysis of optical coherence tomographic angiography (OCT-A) in patients with non-arteritic anterior ischemic optic neuropathy (NAION) corresponds to visual function
Source: PLoS One. 2018 Jun 28;13(6):e0199793. doi: 10.1371/journal.pone.0199793 (PMC6023180; doi:10.1371/journal.pone.0199793)
Supplement: S1 Table — Details regarding the age, gender, onset, visual acuity and automated perimetry performance indices are provided for the 7 patients presenting with acute NAION. (DOCX) [file pone.0199793.s001.docx]

**Supplemental Table 1. Acute NAION cases**

|  | **Age (years)** | **Gender** | **Onset-Presentation (days)** | **Best-corrected visual acuity (Snellen)** | **Mean Deviation (dB)** | **Pattern Standard Deviation (dB)** |
| --- | --- | --- | --- | --- | --- | --- |
| **Patient 1** | 65 | Female | 2 | 20/30-2 | -13.69 | 13.95 |
| **Patient 2** | 42 | Male | 4 | 20/15 | -16.44 | 16.41 |
| **Patient 3** | 67 | Female | 5 | 20/20-1 | -14.56 | 9.66 |
| **Patient 4** | 50 | Male | 48 | 20/125 | -11.22 | 8.27 |
| **Patient 5** | 50 | Male | 9 | 20/50 | -28.45 | 8.32 |
| **Patient 6** | 56 | Male | 7 | 20/15 | -16.05 | 12.10 |
| **Patient 7** | 81 | Male | NA* | 20/25 | -9.66 | 12.56 |

* Onset unknown, optic disc edema was found on routine examination.
