# Supplementary figures and images for: Quantitative analysis of optical coherence tomographic angiography (OCT-A) in patients with non-arteritic anterior ischemic optic neuropathy (NAION) corresponds to visual function
Source: PLoS One. 2018 Jun 28;13(6):e0199793. doi: 10.1371/journal.pone.0199793 (PMC6023180; doi:10.1371/journal.pone.0199793)

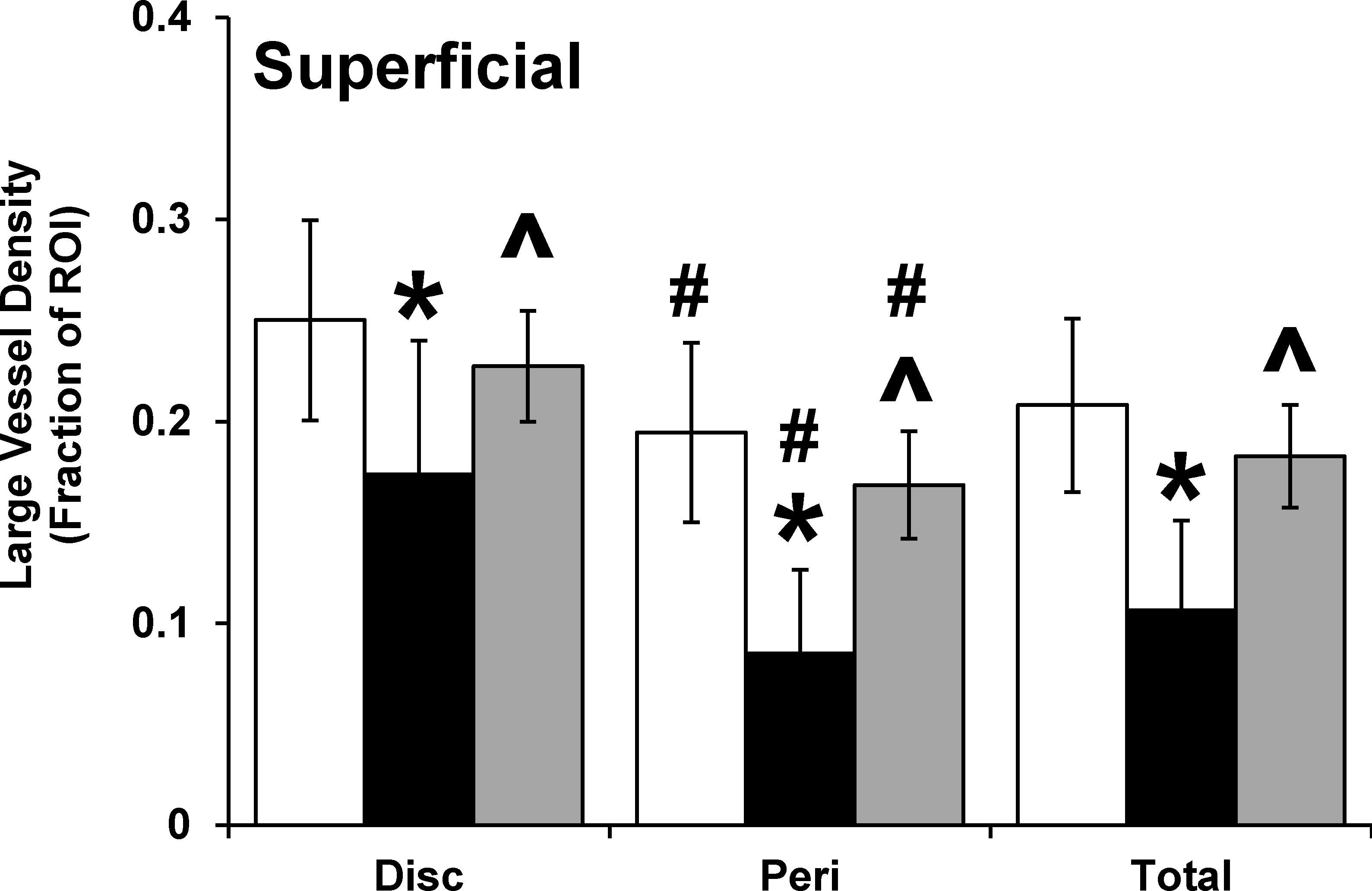

Supplement: S1 Fig — Large, major retinal vessel density was quantified within superficial segmentation lamina and compared between unaffected, acutely affected and non-acutely affected eyes. Error bars represent standard deviation. * denotes p<0.05 versus unaffected; ^ denotes p<0.05 versus the acute group; # denotes p<0.05 versus disc. (TIFF) [file pone.0199793.s002.tiff]

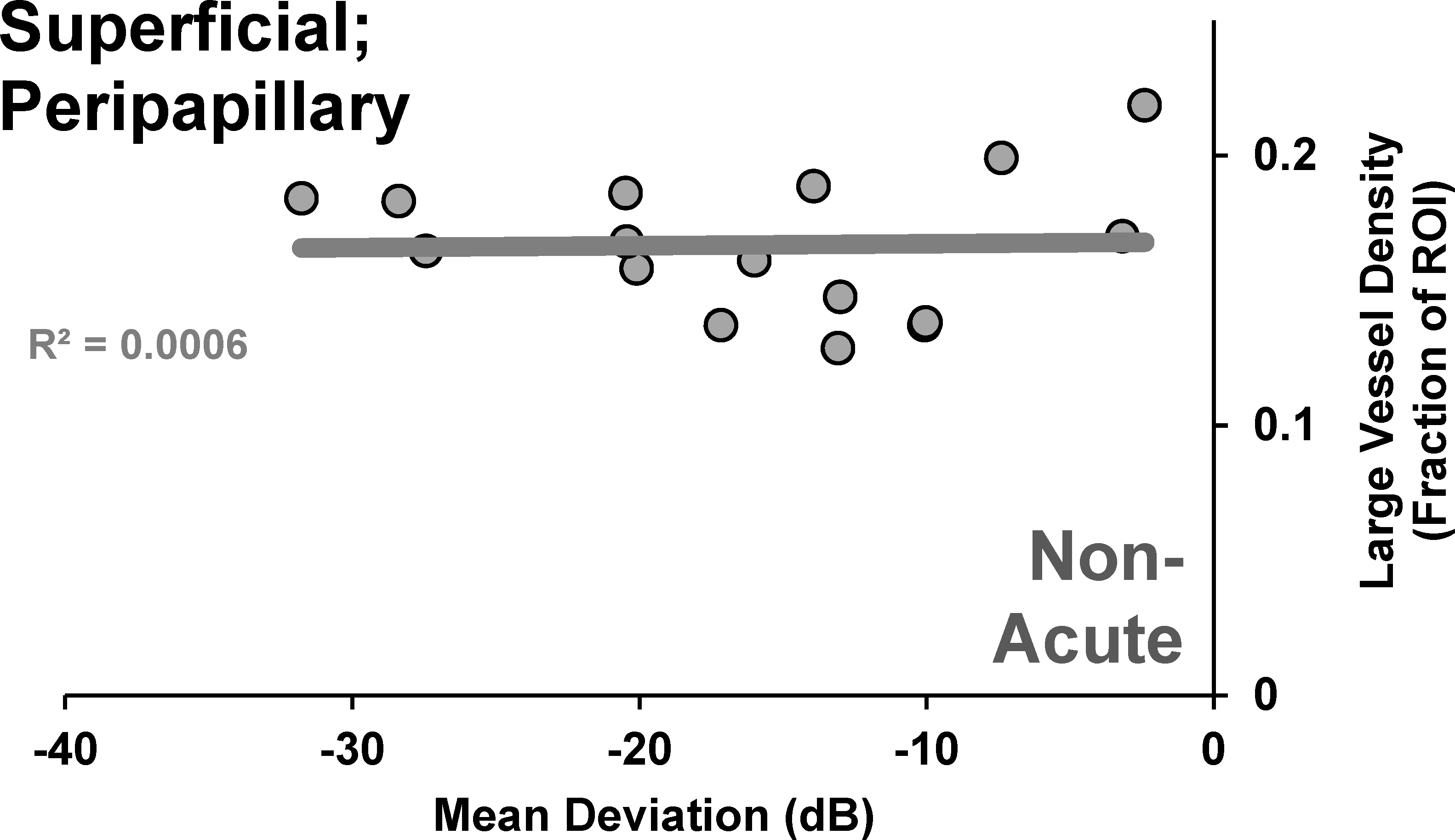

Supplement: S2 Fig — Large, major retinal vessel density in the peripapillary region is plotted according to mean deviation among eyes with non-acute NAION. The trend line represents the linear best fit, and the R2 value is provided. (TIFF) [file pone.0199793.s003.tiff]
